# 

**Published:** 1895-10-26

**Authors:** 


### CONTENTS.

**Sixty Thousand Pounds for the London Hospitals** - 55
**Sewage in Scotch Lochs** - - - - - - - - - 56
**The Diagnosis of Aneurism.** By A. PEARCE GOULD, F.R.C.S. 57
**Modern Sociology.**—Health in the Schoolroom - - - - - 58
**Medical Progress and Hospital Clinics—**
The Causes and Treatment of Ununited Fracture - - - 61
PROGRESS IN MEDICINE.—Graves' Disease - - - - - - 62
PROGRESS IN LARYNGOLOGY (*continued*) - - - - - - - 65
PROGRESS IN RHINOLOGY - - - - - - - - - - - 66
NEW APPLIANCES AND THINGS MEDICAL - - - - - - 66
**Annotations.**—Spurious Hospitals—An Artificial Larynx—The Harveian Oration: Then and Now - - - - - - - - - 59
**Notes and News** - - - - - - - - - - - - 60
**The Metropolitan Hospital Sunday Fund, 1895** - - 67
**The Story of the Insane from Year to Year** - - - 68
**Construction Notes** - - - - - - - - - - - 69
**Scraps and Gleanings** - - - - - - - - - - 69
**The Book World of Medicine and Science** - - - - 70
**The Stock Exchange and the Hospitals** - - - - - 71
**The Student of Medicine** - - - - - - - - - 72

### EXTRA SUPPLEMENT.—THE NURSING MIRROR.

**News from the Nursing World.**—Great Northern Hospital Ladies' Association—Guy's Nurses' Choral Society—Hostel of St. Luke—Exhibition of Nursing Appliances—What are Women Worth?—Nursing at Birkenhead Workhouse—Convalescent Home at New Brighton—District Nursing at Stockport—Promotion for Wardmaids—The Results of the Aberdeen Bazaar—"In this Week's HOSPITAL"—Ballymoney Workhouse Infirmary—Prevention of Cruelty to Children—Short Items - xxv
**Nursing in Rome.** IV.—The Children's Hospital - - - xxvii
**On Urine Testing.**—I. Introductory - - - - - - xxviii
**Elementary Physiology for Nurses.** By C. F. MARSHALL, late Surgical Registrar, Hospital for Sick Children, Great Ormond Street. XII.—The Nervous System - - - xxvii
**Canine Collectors for Hospital Funds** - - - - xxviii
**London Nurses' Training Schools** - - - - - - xxix
**Royal British Nurses' Association** - - - - - - xxx
**Wants and Workers** - - - - - - - - - - - xxxii
**Appointments** - - - - - - - - - - - - - xxxii
**Everybody's Opinion** - - - - - - - - - - - xxxii
**Notes and Queries** - - - - - - - - - - - - xxxii

